# An Essential Membrane Protein Modulates the Proteolysis of LpxC to Control Lipopolysaccharide Synthesis in Escherichia coli

**DOI:** 10.1128/mBio.00939-20

**Published:** 2020-05-19

**Authors:** Elayne M. Fivenson, Thomas G. Bernhardt

**Affiliations:** a Department of Microbiology, Harvard Medical School, Boston, Massachusetts, USA; b Howard Hughes Medical Institute, Harvard Medical School, Boston, Massachusetts, USA; National Cancer Institute

**Keywords:** LpxC, YejM, lipid A, outer membrane, phospholipid

## Abstract

Gram-negative bacteria are surrounded by a complex cell envelope that includes two membranes. The outer membrane prevents many drugs from entering these cells and is thus a major determinant of their intrinsic antibiotic resistance. This barrier function is imparted by the asymmetric architecture of the membrane with lipopolysaccharide (LPS) in the outer leaflet and phospholipids in the inner leaflet. The LPS and phospholipid synthesis pathways share an intermediate. Proper membrane biogenesis therefore requires that the flux through each pathway be balanced. In Escherichia coli, a major control point in establishing this balance is the committed step of LPS synthesis mediated by LpxC. Levels of this enzyme are controlled through its degradation by the inner membrane protease FtsH and its presumed adapter protein LapB (YciM). How turnover of LpxC is controlled has remained unclear for many years. Here, we demonstrate that the essential protein of unknown function YejM (PbgA) participates in this regulatory pathway. Suppressors of YejM essentiality were identified in *lpxC* and *lapB*, and LpxC overproduction was shown to be sufficient to allow survival of Δ*yejM* mutants. Furthermore, the stability of LpxC was shown to be reduced in cells lacking YejM, and genetic and physical interactions between LapB and YejM were detected. Taken together, our results are consistent with a model in which YejM directly modulates LpxC turnover by FtsH-LapB to regulate LPS synthesis and maintain membrane homeostasis.

## Abstract (precis)

The outer membrane is a major determinant of the intrinsic antibiotic resistance of Gram-negative bacteria. It is composed of both lipopolysaccharide (LPS) and phospholipid, and the synthesis of these lipid species must be balanced for the membrane to maintain its barrier function in blocking drug entry. In this study, we identified an essential protein of unknown function as a key new factor in modulating LPS synthesis in the model bacterium Escherichia coli. Our results provide novel insight into how this organism and most likely other Gram-negative bacteria maintain membrane homeostasis and their intrinsic resistance to antibiotics.

## INTRODUCTION

The bacterial cell envelope is essential for maintaining cell shape, withstanding mechanical stress, and resisting osmotic pressure and is a bacterium’s first line of defense against antibiotics, bacteriophages, and immune cells (1, 2). In Gram-negative bacteria, the cell envelope includes a symmetric inner membrane (IM) composed of phospholipids and an asymmetric outer membrane (OM) consisting of phospholipids in the inner leaflet and lipopolysaccharide (LPS) in the outer leaflet (2). The cell wall is located in the periplasmic space between the IM and OM and is made from a cross-linked heteropolymer called peptidoglycan (PG). Biogenesis of the cell envelope is tightly coordinated with cellular growth and division (3), with all three layers having to expand and divide each cell cycle without compromising their structural integrity.

The phospholipid (PL) and LPS membrane components are synthesized in the cytosol and within the inner leaflet of the IM. They must be transported across the IM and through the periplasm to build the OM (for reviews, see references 1 and 4–6). LPS consists of three covalently attached groups: a glycolipid called lipid A, a core oligosaccharide, and a longer, variable O-antigen polysaccharide chain. O antigen is synthesized separately from the lipid A core, but the two components are joined by ligation while anchored in the outer leaflet of the IM. Mature LPS molecules are then transported from the IM to the OM by the Lpt system, which forms a protein bridge connecting the IM and OM (7). The mechanism by which PLs are transported to the OM remains unclear (8).

**FIG 1:**
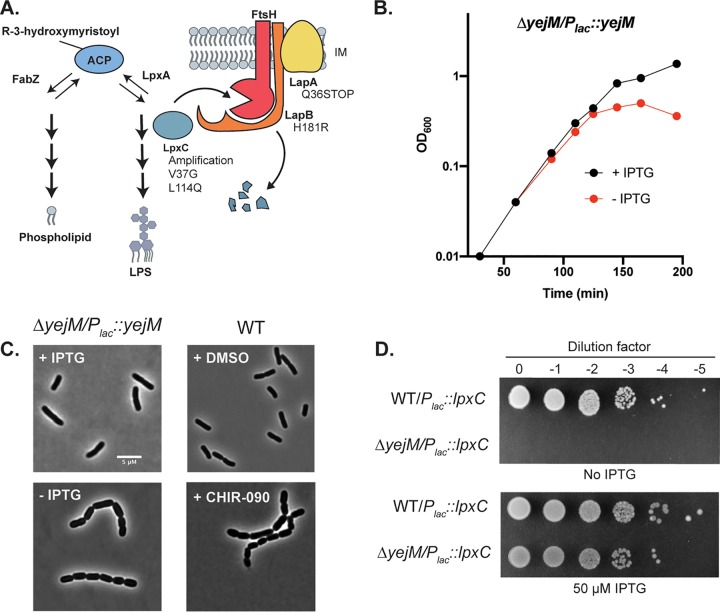
The depletion of YejM leads to cell chaining and lysis. (A) Branched synthesis pathway leading from *R*-3-hydroxymyistoyl-ACP to either phospholipid or LPS. LpxC is turned over by FtsH. LapB is thought to serve as an adapter in this process. LapA is also shown, but its role in LpxC degradation is not clear. The identities of variants that suppress YejM essentiality are indicated in the diagram. (B) Growth curve following the depletion of YejM. Cells of strain EMF27 (*ΔyejM*/P*_lac_*::*yejM*) were grown without (red) or with (black) 50 μM IPTG to induce *yejM* expression, and growth was monitored by following the OD_600_ of the culture. (C) (Left) Micrographs of cells at the 125-min time point of the growth curve shown in panel B. (Right) Wild-type cells treated with DMSO (top) or 0.25 μg/ml CHIR-090 (bottom) for 2 h. Bar, 5 μm. (D) Serial dilutions of wild-type and *ΔyejM* cells harboring a P*_lac_*::*lpxC* plasmid were plated in the absence and presence of 50 μM IPTG.

*R*-3-Hydroxymyristoyl-acyl carrier protein (ACP) serves as the acyl donor for the synthesis of both PL and LPS (Fig. 1A). In the PL synthesis pathway, it is a substrate of (3*R*)-hydroxymyristoyl-ACP dehydratase (FabZ) (9). It is also utilized by LpxA (UDP-*N*-acetylglucosamine acyltransferase) in the first step of lipid A synthesis (10). However, this reaction is reversible (11); the committed step for lipid A production is catalyzed by the second enzyme, UDP-3-*O*-acyl-*N*-acetylglucosamine deacetylase (LpxC) (12). Balanced synthesis of LPS and PL is required to prevent loss of membrane integrity and cell death (13). In Escherichia coli, this balance is in part maintained by the inner membrane-localized, ATP-dependent zinc metalloprotease FtsH (14). In conjunction with its presumed adapter protein LapB (also called YciM) (15, 16), FtsH degrades LpxC to regulate the flux of lipid precursors through the LPS pathway. However, how LpxC proteolysis is modulated in response to disruptions in LPS or PL synthesis to maintain homeostasis is unclear.

Here, we identified the essential membrane protein of unknown function YejM (also called PbgA) as an inhibitor of LpxC turnover by FtsH-LapB in E. coli. Genetic suppressors of *yejM* essentiality first pointed us toward its potential role in maintaining sufficient LpxC levels for LPS synthesis. Subsequent analysis indicated that LpxC stability is reduced in the absence of YejM and that YejM interacts genetically and physically with LapB. Our results are therefore consistent with a model in which YejM interferes with LpxC proteolysis through its interaction with LapB. Complementary results that also support this model were reported while this paper was under review (17). We propose that the modulation of LpxC degradation by YejM is likely to be homeostatic and responsive to perturbations in the balance between LPS and PL synthesis.

## RESULTS

### Rationale.

In a genetic selection for suppressors of cell morphogenesis defects in E. coli, we identified mutations in *yejM*. These suppressors will be reported as part of a separate study, but they prompted us to further investigate YejM (PbgA) function, especially because it has remained one of the few essential genes in E. coli without a well-characterized activity. YejM is an IM protein with a five-transmembrane-domain N terminus that is essential for growth and a nonessential C-terminal periplasmic domain (18, 19). Nonsense mutations in *yejM* leading to the truncation of the periplasmic domain have previously been found to cause phenotypes consistent with defects in OM assembly (18–21), including reduced LPS/PL ratio, vancomycin sensitivity, temperature sensitivity, and leakage of periplasmic proteins. YejM shares structural similarities to LtaS, the enzyme that synthesizes lipoteichoic acids in many Gram-positive bacteria (22). Like LtaS, YejM has a hydrophobic binding pocket in the periplasmic domain that is important for protein function (23). However, the crystal structure of this domain of YejM revealed that it lacks residues that are important for LtaS catalytic activity, indicating that is unlikely to have a similar enzymatic function (23). Although previous studies have implicated YejM in the transport of cardiolipin to the OM in Salmonella enterica serovar Typhimurium (24, 25) and Shigella flexneri (26), a recent study suggested that it plays a broader yet ill-defined role in envelope assembly (27). We therefore thought that further study of the function of this essential protein was warranted.

### Overproduction of LpxC suppresses the essentiality of YejM.

To begin investigating the essential function of YejM, we examined the terminal morphological phenotype induced upon its depletion. The native *yejM* gene was deleted in a strain harboring a plasmid that expressed *yejM* from an IPTG (isopropyl-β-d-thiogalactopyranoside)-inducible *lac* promoter (P*_lac_*). In the presence of inducer, these cells grew and divided normally (Fig. 1B). However, as observed previously (18, 19, 24), YejM depletion in the absence of IPTG led to slowed growth followed by partial lysis of the culture (Fig. 1B). Prior to lysing, the YejM-depleted cells formed cell chains (Fig. 1C; also, see Fig. S1 in the supplemental material), indicating a failure to complete cell separation. This morphology is reminiscent of cells defective in envelope biogenesis, including mutants defective in LPS assembly (28–30) and cells treated with the LpxC inhibitor CHIR-090 (Fig. 1C; also, see Fig. S1).

10.1128/mBio.00939-20.1 FIG S1 Quantification of cell chaining in the absence of YejM or following treatment with LpxC inhibitor CHIR-090. Cell chaining (Fig. 1C) was quantified by counting the number of cellular units per cell following the depletion of YejM (*n* = 20) (A) or treatment with 0.25 μg/ml CHIR-090 for 2 h (*n* =45) (B). The data were then plotted in Graphpad Prism. ****, *P < *0.0001 (unpaired t-test). Download FIG S1, TIF file, 0.6 MB. Copyright © 2020 Fivenson and Bernhardt. 2020 Fivenson and Bernhardt This content is distributed under the terms of the Creative Commons Attribution 4.0 International license.

We next turned to suppressor analysis to identify mutations that bypass the essentiality of YejM as a means to understand its function. To this end, the *yejM* gene was cloned under the control of the lactose promoter (P*_lac_*) in a plasmid backbone designed by the de Boer laboratory to select for suppressors of *ftsN* essentiality (31). The vector encodes a temperature-sensitive replication protein [RepA(Ts)] and the restriction endonuclease I-SceI under the control of a temperature-sensitive lambda repressor (*c*I857), which is paired with an I-SceI cutting site in the vector backbone. To select for suppressors of YejM essentiality, a Δ*yejM* strain harboring the *yejM* suicide vector was plated at the nonpermissive temperature (37°C), where the plasmid will cease to replicate and be cleaved by expressed I-SceI. Under these conditions, suppressors arose at a frequency of ∼9 × 10^−4^. To increase the stringency of the selection, suppressors were also isolated on LB plates containing 1% sodium dodecyl sulfate (SDS), which reduced the suppressor frequency to ∼1 × 10^−5^. Twelve surviving colonies (5 selected on LB and 7 selected on LB containing 1% SDS) were isolated, and their mutations were mapped by whole-genome sequencing (Fig. 1A; also, see Table S1). Five of the suppressors contained genomic amplifications in a region containing *lpxC*. Two additional suppressors had missense mutations in *lpxC*, including one encoding a V37G substitution that has been demonstrated to lead to increased LpxC abundance in Klebsiella pneumoniae, suggesting increased stability (32). Three of the suppressors had mutations either in *lapB*, in its neighbor *lapA*, or in the region upstream of the *lapAB* operon. Overall, 10 of 12 suppressors had mutations predicted to increase cellular LpxC levels.

10.1128/mBio.00939-20.4 TABLE S1 *ΔyejM* suppressors. Mutations mapped in each suppressor are listed. Download Table S1, PDF file, 0.1 MB. Copyright © 2020 Fivenson and Bernhardt. 2020 Fivenson and Bernhardt This content is distributed under the terms of the Creative Commons Attribution 4.0 International license.

The suppressor analysis suggested that an LpxC deficiency underpinned the lethality of a Δ*yejM* mutation. To investigate this possibility further, *lpxC* was cloned under P*_lac_* control on a multicopy plasmid. A strain harboring this vector was used as a recipient in a transduction using P1 phage grown on a Δ*yejM*::*kan*^r^ donor strain. Kanamycin-resistant transductants were readily obtained on agar containing IPTG to induce *lpxC* expression from the plasmid, and these strains were found to be inducer-dependent for growth (Fig. 1D). Notably, the only previously reported suppressor of a Δ*yejM* mutation was a multicopy vector encoding holo-ACP synthase 2 (AcpT) (18–20), and catalytic activity of the synthase was found not to be required for suppression (18). Given our results, we wondered whether overproduction of AcpT also acts by promoting the accumulation of LpxC. Cells overexpressing *acpT* from a plasmid were indeed found to have elevated LpxC levels (see Fig. S2), suggesting that elevated AcpT may be an FtsH substrate and overwhelm the proteolytic machinery to allow LpxC accumulation. Taken together, our results thus far indicate that LpxC overproduction renders the normally essential YejM protein dispensable for growth.

10.1128/mBio.00939-20.2 FIG S2 The overexpression of *acpT* increases LpxC levels. Immunoblot detection of LpxC from extracts of wild-type cells harboring plasmids pPR111 (P*_lac_*::*lpxC*), pPR66 (P*_lac_*::empty), or pEMF43 (P*_lac_*::*acpT*) grown in the presence or absence of IPTG (50 μM [lane 2] or 1 mM [lanes 4 and 6]), as indicated. Download FIG S2, TIF file, 0.6 MB. Copyright © 2020 Fivenson and Bernhardt. 2020 Fivenson and Bernhardt This content is distributed under the terms of the Creative Commons Attribution 4.0 International license.

### LpxC is aberrantly degraded in the absence of YejM.

**FIG 2:**
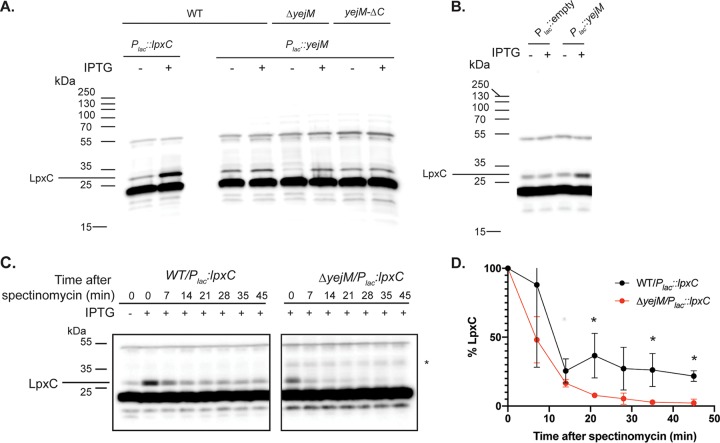
Changes in *yejM* expression affect LpxC accumulation. (A) Immunoblot using anti-LpxC primary antibody. Lanes 1 and 2, extracts from wild-type cells with a P*_lac_*::*lpxC* (pPR111) plasmid grown in the absence (lane 1) or presence (lane 2) of 50 μM IPTG to serve as a marker for LpxC; lanes 3 and 4, wild-type cells with a P*_lac_*::*yejM* (pEMF17) plasmid grown for 195 min in the absence (lane 3) or presence (lane 4) of 50 μM IPTG; lanes 5 and 6, extracts from *ΔyejM* cells with a P*_lac_*::*yejM* (pEMF17) plasmid grown for 195 min in the absence (lane 5) or presence (lane 6) of 50 μM IPTG; lanes 7 and 8, extracts from *yejM-ΔC* cells with a P*_lac_*::*yejM* (pEMF17) plasmid grown for 195 min in the absence (lane 7) or presence (lane 8) of 50 μM IPTG. (B) Immunoblot detecting LpxC from extracts of wild-type cells with a P*_lac_*::empty (pPR66) or a P*_lac_*::*artificialRBS_yejM* (pEMF33) plasmid grown in the absence (lanes 1 and 3) or presence (lanes 2 and 4) of 1 mM IPTG. Note that the *yejM* in this plasmid has a strong artificial ribosome-binding site relative to the construct used for panel A. (C) Wild-type and *ΔyejM* cells harboring a P*_lac_*::*lpxC* plasmid (pPR111) were grown in the presence of 50 μM IPTG at 30°C in minimal medium to an OD_600_ of 0.5. Spectinomycin (300 μg/ml) was then added, and samples were taken at the indicated time points for the preparation of extracts and the detection of LpxC by immunoblotting. The asterisk denotes a nonspecific band that appeared sporadically in blots of the *ΔyejM* samples. Blots are representative of three independent experiments. Note that the first lane of the left blot shows the LpxC level in uninduced cells. (D) Quantification of LpxC levels following spectinomycin treatment in three independent experiments. Levels of LpxC are presented as a percentage of the initial LpxC concentration at time zero. The asterisk indicates a *P* value of <0.05 (unpaired *t* test). Error bars denote standard deviations (SD).

To further investigate the connection between YejM and LpxC, we investigated the effect of YejM inactivation on the steady-state levels of LpxC. Immunoblotting indicated that LpxC levels were unaffected in wild-type cells with or without moderate induction of *yejM* expression from a plasmid (Fig. 2A). However, depletion of YejM in cells with a deletion of the native gene led to a dramatic reduction in LpxC levels, to the point where it was barely detectable (Fig. 2A). Notably, cells harboring a *yejM* allele (*yejM-ΔC*) at its native locus in which a stop codon was introduced at residue 192 maintained normal LpxC levels whether or not expression of the full-length gene was induced from a plasmid (Fig. 2A), indicating that the N-terminal transmembrane domain of YejM is sufficient to prevent aberrant degradation of LpxC. Furthermore, increased expression of *yejM* from a plasmid with higher concentrations of inducer led to a striking increase in LpxC accumulation in otherwise wild-type cells (Fig. 2B). Based on these results, we hypothesized that LpxC is aberrantly degraded in the absence of YejM. To test this possibility, we took advantage of our ability to delete *yejM* in the presence of a plasmid overproducing *lpxC*. Wild-type and Δ*yejM* cells expressing *lpxC* from the plasmid were treated with spectinomycin to block translation, and the fate of previously translated LpxC was monitored by immunoblotting. Because LpxC is initially overproduced, rapid degradation of the protein is observed in both cell types in the first 7 min, which presumably reflects the degradation system attempting to return the concentration of LpxC to endogenous (uninduced) levels. After the initial phase of degradation, the LpxC concentration plateaued in wild-type cells at about 25% of the initial concentration and was maintained at this level for the rest of the time course (Fig. 2C). In contrast, LpxC levels decayed rapidly in the Δ*yejM* cells to undetectable levels without an observable plateau (Fig. 2C). Based on these results, we conclude that YejM is required to prevent excessive turnover of LpxC.

### YejM interacts genetically and physically with LapB.

**FIG 3:**
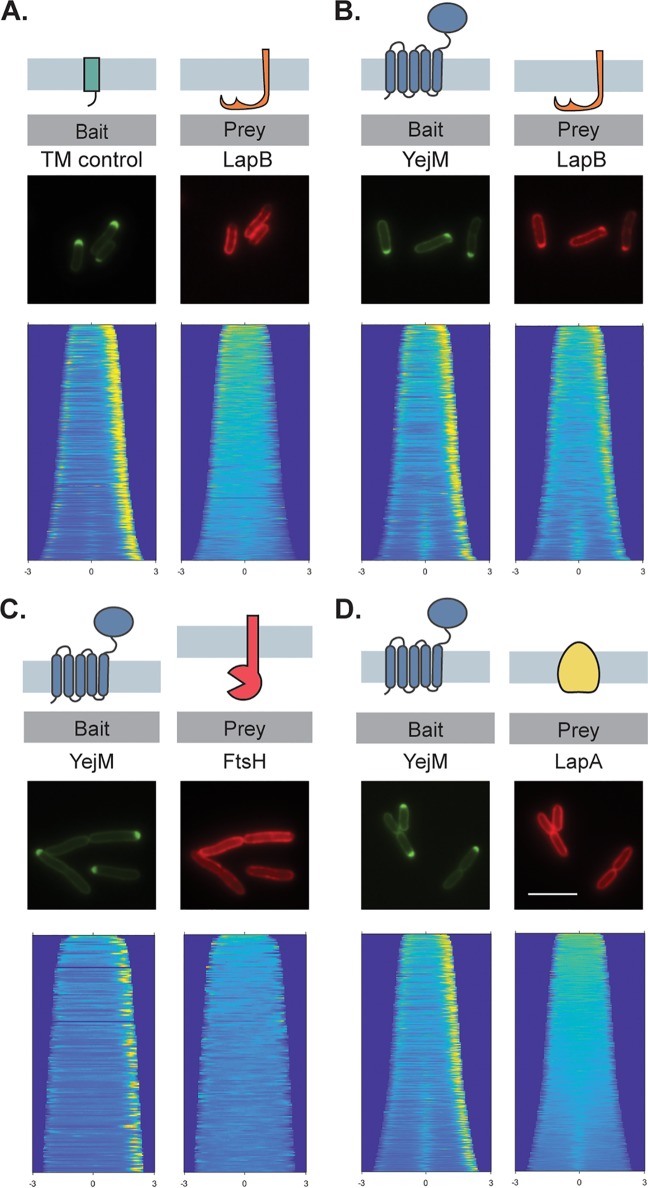
YejM interacts with LapB. The POLAR assay was used to assess protein-protein interactions (see the text for details). Shown are representative micrographs of cells expressing the indicated bait and prey proteins. Cells were transformed with plasmids producing the control bait, which consists of a single transmembrane domain derived from residues 2 to 55 of Pseudomonas aeruginosa PBP1b fused to PopZ-H3H4-GFP (pEMF55) (A) or PopZ-H3H4-GFP-YejM (pEMF35) (B to D). Note that the control bait construct also expresses unlabeled *yejM* in order to reduce LapB toxicity. Prey constructs produce C-terminal mScarlet fusions to the indicated proteins from plasmid vectors integrated into the chromosome. Bar, 5 μm.

Our results thus far suggested a model in which YejM promotes LpxC accumulation by protecting it from the FtsH-LapB proteolytic system. We therefore investigated whether YejM interacts with any of the components of this system using our recently developed POLAR (PopZ-linked apical recruitment) two-hybrid assay (33). The POLAR assay takes advantage of the ability of the PopZ protein from Caulobacter crescentus to spontaneously form foci at the poles of E. coli cells. Therefore, protein-protein interactions can be assessed by fusing a “bait” protein to a PopZ-interaction domain called H3H4 along with green fluorescent protein (GFP) and then monitoring whether it can recruit a “prey” protein of interest fused to mScarlet to the cell pole. Using YejM as a bait, we found that it was able to recruit a LapB prey fusion to the pole (Fig. 3B), but not an FtsH or a LapA prey fusion (Fig. 3C and D). The recruitment of LapB prey to the pole was specific for YejM, as it was not recruited by a transmembrane control bait (Fig. 3A). However, we noticed that cells expressing LapB prey constructs formed chains and appeared to lyse when they were paired with the control bait (see Fig. S3A) but not with the YejM bait. Therefore, for the control in Fig. 3A we used a control bait construct in which *yejM* was silently expressed downstream of the control bait sequence, which eliminated the adverse morphological effects observed upon production of the LapB prey fusion. We also observed a positive interaction between YejM-ΔC and LapB (Fig. S3B), but unlike in assays with the full-length YejM bait and LapB prey, cell chaining was observed. Thus, some residual toxicity of the LapB prey construct was likely maintained when it was paired with the YejM-ΔC bait. Based on the POLAR results, we conclude that YejM interacts with the LapB component of the FtsH-LapB proteolytic system and that the N-terminal transmembrane domain of YejM is sufficient for this interaction.

10.1128/mBio.00939-20.3 FIG S3 The LapB prey construct does not interact with the control bait construct in the POLAR assay. Shown are representative micrographs of cells expressing the indicated bait and prey proteins. (A) Cells were transformed with plasmids expressing the control bait, which consists of a single transmembrane domain derived from residues 2 to 55 of Pseudomonas aeruginosa PBP1b fused to PopZ-H3H4-GFP (pHCL149). The LapB prey construct consists of a C-terminal mScarlet fusion to LapB and was expressed from an integrated plasmid (pHCL147 derivative pEMF36) under the control of the P*_lac_* promoter. The experimental setup is the same as for Fig. 3A, except that unlabeled YejM is not produced from the bait construct in this case. Note that without expressing the unlabeled YejM, cells begin to form chains, most likely due to the toxic effects of LapB overproduction. (B) The LapB prey construct is coexpressed with the YejM-ΔC bait construct (pEMF65). Bar, 5 μm. Download FIG S3, TIF file, 1.0 MB. Copyright © 2020 Fivenson and Bernhardt. 2020 Fivenson and Bernhardt This content is distributed under the terms of the Creative Commons Attribution 4.0 International license.

**FIG 4:**
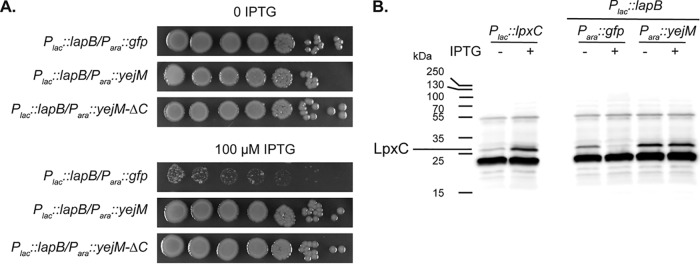
YejM protects cells from LapB toxicity. (A) Serial dilutions of wild-type cells with the integrated *lapB* overexpression plasmid pEMF53 (P*_lac_*::*lapB*) and either pEMF57 (P*_ara_*::*gfp*), pEMF54 (P*_ara_*::*yejM*), or pEMF68 (P*_ara_*::*yejM-ΔC*) were plated on LB agar with 0.2% arabinose and additionally supplemented with 100 μM IPTG, as indicated. Note that *lapB*-induced toxicity is relieved by *yejM* coexpression. (B) LpxC immunoblot. Extracts from WT cells harboring a P*_lac_*::*lpxC* plasmid (pPR111) grown with (lane 2) or without (lane 1) IPTG were used as a marker for the LpxC band. LpxC was also detected in extracts of MG1655(attHKEMF53) [WT(P*_lac_*::*lapB*)] cells harboring either pEMF57 (P*_ara_*::*gfp*) (lanes 4 and 5) or pEMF54 (P*_ara_*::*yejM*) (lanes 6 and 7) grown in LB containing 0.2% arabinose without (lanes 4 and 6) or with (lanes 5 and 7) 100 μM IPTG to induce *lapB* expression.

The effects of the LapB prey fusion observed on cell growth during the POLAR analysis suggested that LapB overproduction is toxic and that this toxicity can be overcome by coexpression of *yejM*. To investigate this possibility further, we monitored the effect of overproducing untagged LapB on cell growth when either full-length YejM, YejM-ΔC, or a GFP control was simultaneously overproduced from a compatible plasmid. Cells containing the *lapB* expression plasmid grew normally in the absence of inducer regardless of whether they overproduced YejM or GFP (Fig. 4A). When *lapB* expression was induced, plating efficiency was dramatically reduced for cells co-producing GFP (Fig. 4A). However, cells coproducing full-length YejM or YejM-ΔC were protected and plated at normal efficiency (Fig. 4A). To investigate whether the toxicity of LapB and its antagonism by YejM were related to LpxC turnover, we monitored LpxC abundance in cells overproducing LapB. Cells in which LapB and GFP were coproduced had reduced LpxC levels, whereas cells overexpressing *yejM* had elevated levels of LpxC and no apparent reduction in LpxC levels upon LapB overproduction (Fig. 4B). We conclude not only that YejM interacts with LapB but also that it likely serves as an antagonist of LapB activity to prevent LpxC degradation by the FtsH-LapB proteolytic machinery.

## DISCUSSION

YejM (PbgA) has remained one of the few essential proteins of unknown function in E. coli. With the exception of a multicopy suppressor selection (18, 19), prior genetic studies primarily examined the phenotype of mutants encoding a truncated YejM protein lacking the nonessential C-terminal periplasmic domain (18–21, 24, 27). Cells producing these YejM-ΔC variants displayed a range of OM permeability barrier defects, suggesting an important yet ill-defined role for YejM in envelope biogenesis.

A more specific role was assigned to the YejM homolog from *S.* Typhimurium (PbgA), which was identified as a factor required for OM barrier function in cells activated for the two-component system PhoPQ (24). This regulatory system induces changes in the OM that help protect *S.* Typhimurium from the assaults it encounters in the phagosomes of host cells during infection (34). One of the observed changes in OM composition during PhoPQ induction is an increase in the content of the phospholipid cardiolipin (CL) (24, 35). This increase in CL levels in the OM was not observed in PhoPQ-activated cells producing a YejM-ΔC variant. This observation combined with the detection of CL binding by the YejM periplasmic domain *in vitro* led to the original proposal that the protein functions as a transporter that shuttles CL from the IM to the OM (24). A structure of full-length YejM was recently reported in which the protein was premixed with cardiolipin prior to crystallization (25). Two cardiolipin binding sites were observed in the structure, but they were positioned near the membrane facing surface of the transmembrane domain where any phospholipid species might be expected to interact with YejM when it is situated in the IM (25). Thus, the new structural information does not help explain how YejM might function as a cardiolipin transporter.

In another recent follow-up to the original *S.* Typhimurium study (24), mice were infected with *S.* Typhimurium producing a YejM-ΔC variant, and following growth in the host, suppressors that restored OM integrity to the YejM-ΔC cells were isolated in *lpxC*, *lapB*, and *ftsH* (27). It was also found that deletion of the C-terminal domain of YejM resulted in changes in the LPS and phospholipid composition of *S.* Typhimurium that could be at least partially rescued by the suppressors (27). As a result of this analysis, a variety of functions were proposed for YejM, including a general role in LPS assembly. It was also proposed that the periplasmic domain of YejM somehow facilitates lipid trafficking during stress and that the transmembrane region performs an undefined essential activity involving phospholipids (27).

**FIG 5:**
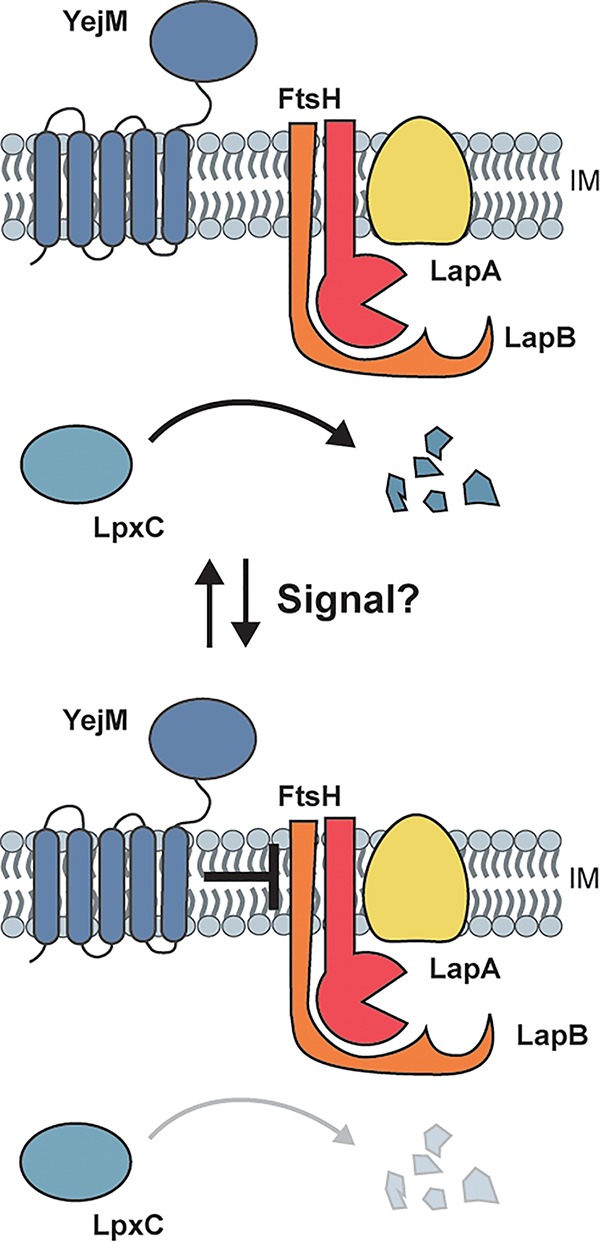
Model for the modulation of LpxC turnover by YejM. LpxC is degraded by FtsH in a LapB-dependent manner. The role of LapA is unclear. We propose that in response to some signal, potentially the buildup of a lipid molecule in the inner membrane, YejM blocks LpxC degradation by FtsH-LapB. This inhibition is most likely mediated by an interaction between YejM and LapB and may be used to help balance LPS and phospholipid synthesis in response to perturbations or fluctuations.

A simplified picture of YejM function emerges from our study of a complete deletion allele of *yejM* in E. coli. Similar to the host-induced suppressors of the *S.* Typhimurium YejM-ΔC defect, we identified suppressors of YejM essentiality in the *lpxC* and *lapB* genes. We further showed that overexpression of *lpxC* was sufficient to allow complete deletion of *yejM*. Subsequent analysis demonstrated that levels of LpxC were strongly dependent on YejM. Inactivation of YejM led to aberrant degradation of LpxC and reduced levels of the enzyme, whereas overproduction of YejM promoted the hyperaccumulation of LpxC. We then identified an interaction between YejM and LapB, likely mediated through the N-terminal transmembrane domain of YejM, and discovered that the toxicity of LapB overproduction can be blocked by co-overproduction of either full-length YejM or YejM-ΔC. Overall, our results are consistent with a model in which YejM opposes LapB function to inhibit LpxC degradation by the FtsH protease (Fig. 5).

The ability of YejM to antagonize LpxC degradation makes it an attractive candidate for a factor that modulates the stability of LpxC in response to disruptions in LPS and phospholipid synthesis homeostasis. It has been known for some time that inhibition of LpxC or overexpression of *fabZ*, both of which presumably shift flux of lipid precursors away from the LPS synthesis pathway, leads to the stabilization of LpxC (9, 32, 36–38). Recently, it was also shown that LpxC turnover is reduced in cells with elevated activity of phospholipase A (PldA), which cleaves phospholipids in the outer leaflet of the OM, another marker for defects in LPS and phospholipid synthesis homeostasis (39). In each of these cases, the precise molecular signal(s) that modulates LpxC degradation by FtsH-LapB remains to be determined. However, lipids like acyl coenzyme A (acyl-CoA), LPS, and phospholipids, or intermediates in the synthesis of these molecules, are likely candidates (36, 39). Notably, YejM is related to enzymes like LtaS and EptA that use phospholipid substrates either to polymerize lipoteichoic acids or to modify lipid A with a lipid head group, respectively (22, 40–43). Even though YejM lacks amino acids predicted to be required for enzymatic activity, it is conceivable that it retains the ability to bind LPS, phospholipids, or both, and that such binding events modulate its ability to interfere with LpxC degradation by FtsH-LapB. The C terminus of YejM is likely involved in recognizing any potential signaling molecules despite its dispensability for interacting with LapB or in maintaining normal steady-state levels of LpxC. We therefore suspect that most of the OM defects observed previously for cells producing YejM-ΔC variants, including reduced cardiolipin content in the OM of *S.* Typhimurium, ultimately stem from the improper control of LpxC turnover in response to stress and that a lipid transport function for YejM is unlikely. Although further studies are required to test these possibilities, the identification of YejM as an essential component in the pathway controlling LpxC levels represents an important step toward a mechanistic understanding of how Gram-negative bacteria balance phospholipid and LPS synthesis to properly assemble the OM layer that defines them.

## MATERIALS AND METHODS

### Bacterial strains and growth conditions.

All strains used and generated here are derivatives of MG1655. Strains were cultured in LB medium (1% tryptone, 0.5% yeast extract, 0.5% NaCl) or minimal M9 medium (44) supplemented with 0.2% Casamino Acids and 0.2% glucose, arabinose, or maltose, as indicated in the figure legends.

Antibiotic concentrations are as follows (unless otherwise indicated): 25 μg/ml chloramphenicol (Cam), 25 μg/ml kanamycin (Kan), 5 μg/ml tetracycline (Tet), and 50 μg/ml spectinomycin (Spec). Strains EMF27 [*ΔyejM*::*kan*^r^/P*_lac_*::*yejM*] and EMF30 [*ΔyejM*::*kan^r^*/P*_lac_*::*lpxC*] were maintained on medium supplemented with 50 μM IPTG unless otherwise stated. All strains, plasmids, and primers used in this study are listed in Table S2, Table S3, and Table S4, respectively. Methods used to construct the *ΔyejM* strains and expression constructs used in this study are detailed in the supplemental material.

10.1128/mBio.00939-20.5 TABLE S2 Strains used in this study. Download Table S2, PDF file, 0.04 MB. Copyright © 2020 Fivenson and Bernhardt. 2020 Fivenson and Bernhardt This content is distributed under the terms of the Creative Commons Attribution 4.0 International license.

10.1128/mBio.00939-20.6 TABLE S3 Plasmids used in this study. Download Table S3, PDF file, 0.1 MB. Copyright © 2020 Fivenson and Bernhardt. 2020 Fivenson and Bernhardt This content is distributed under the terms of the Creative Commons Attribution 4.0 International license.

10.1128/mBio.00939-20.7 TABLE S4 Primers used in this study. Download Table S4, PDF file, 0.03 MB. Copyright © 2020 Fivenson and Bernhardt. 2020 Fivenson and Bernhardt This content is distributed under the terms of the Creative Commons Attribution 4.0 International license.

### Selection of suppressors of YejM essentiality.

The suppressor strain plasmid (pEMF20) was cloned via Gibson assembly in strain JLB45, which expresses *c*I857, in order to prevent zygotic induction of I-SceI. The plasmid was then transformed into MG1655 Chung competent cells. The *ΔyejM*::*kan*^r^ allele was transduced into MG1655/pEMF20 via P1 transduction and confirmed via PCR (see Text S1 in the supplemental material), generating strain EMF37. Suppressors of *ΔyejM* were selected by growing EMF37 cells at 37°C on LB plates (including 1% SDS, where indicated). An overnight culture of EMF37 was prepared under the permissive condition (30°C; LB containing Kan, Spec, and 100 μM IPTG). For suppressors EMF40 to -44, the EMF37 overnight culture was serially diluted, plated on LB plates, and incubated at 37°C. For suppressors EMF45 to -51, the overnight culture of EMF37 was back diluted 1:50 and grown under permissive conditions until an optical density at 600 nm (OD_600_) of 0.42 was reached. Cells were then pelleted, washed, and resuspended in LB and allowed to grow for an additional hour at 37°C (nonpermissive conditions). Cells were then plated on LB + 1% SDS and incubated overnight at 37°C. The loss of plasmid pEMF20 was confirmed by screening for spectinomycin sensitivity. Overnight cultures of the suppressor strains were prepared, and 5 ml of each culture was pelleted and stored at −20°C. Genomic DNA (gDNA) was isolated from each pellet using the Wizard genomic DNA purification kit (Promega) and further purified using the genomic DNA Clean & Concentrate kit (Zymo Research). Whole-genome sequencing was performed as described previously (45) with some modifications (46) (Nextera DNA sample preparation kit). The concentration of the DNA in the samples was determined using the Qubit dsDNA HS assay kit, and the sizes of the products following tagmentation were determined using a high-sensitivity D1000 screen tape run on an Agilent 4200 TapeStation. The sequencing was carried out using a MiSeq reagent kit v3 (Illumina). The data were analyzed using the CLC Genomics Workbench software (Qiagen).

10.1128/mBio.00939-20.8 TEXT S1 Supplemental methods for plasmid and strain construction. Download Text S1, PDF file, 0.1 MB. Copyright © 2020 Fivenson and Bernhardt. 2020 Fivenson and Bernhardt This content is distributed under the terms of the Creative Commons Attribution 4.0 International license.

### Immunoblotting.

Cell pellets were collected and resuspended in water and 2× Laemmli sample buffer (100 mM Tris-HCl, pH 6.8; 2% SDS; 0.1% bromophenol blue; 20% glycerol) at a 1:1 ratio. Samples were boiled for 10 min and sonicated (Qsonica tip sonicator; amplification, 25%; time, 1 min) two or three times. Protein concentration was measured using the noninterfering (NI) protein assay (with bovine serum albumin [BSA] protein standard) (G Biosciences catalog no. 786-005) and was normalized using 1× sample buffer. Samples were run on a 15% polyacrylamide gel and transferred to a polyvinylidene difluoride (PVDF) membrane. The membrane was then rinsed in phosphate-buffered saline containing 0.1% Tween (PBS-T) (10% 10× PBS-T buffer, pH 7.4 [Sigma-Aldrich]) and blocked in 5% milk in PBS-T for 1.5 h. The membrane was then incubated in a primary antibody solution of 1% milk in PBS-T containing rabbit anti-lpxC antibody (a generous gift from the Doerrler lab) at a 1:10,000 dilution for approximately 16 h at 4°C. The membrane was then washed four times in PBS-T (once quickly and three times for 10 min each). The membrane was then incubated in secondary antibody solution (horseradish peroxidase [HRP]-conjugated anti-rabbit IgG; 1:1,000 dilution; Rockland no. 18–8816-33) in 0.2% milk in PBS-T for 2 h. Following 5 washes with PBS-T, the membrane was developed using SuperSignal West Pico Plus chemiluminescent substrate (Thermo Fisher Scientific catalog no. 34577) and imaged using the c600 Azure Biosystems platform.

### LpxC degradation assay.

MG1655/pPR111 (wild type [WT]/P*_lac_*::*lpxC*] and EMF30 [Δ*yejM*/P*_lac_*::*lpxC*] cells were incubated overnight in 4 ml of LB containing Cam without or with 50 μM IPTG, respectively. Overnight cultures were diluted to an OD_600_ of 0.025 in 5 ml of LB containing Cam with (MG1655/pPR111 and EMF30) or without (MG1655/pPR111) 50 μM IPTG. Cultures were incubated at 37°C with shaking to an OD_600_ of ∼0.3 to 0.4. Cell concentrations were normalized, and cultures were back diluted again at a ratio of 1:50 in 100 ml M9 minimal medium containing 50 μg/ml chloramphenicol and 50 μM IPTG (except for the MG1655/pPR111 control without IPTG). Cells were grown at 30°C with shaking to an OD_600_ of 0.5. Then, 300 μg/ml spectinomycin was added to the cultures to inhibit protein synthesis. Samples were taken at 0, 7, 14, 21, 28, 35, and 45 min following the addition of spectinomycin and analyzed via immunoblotting for LpxC. The amount of LpxC was quantified by measuring the intensity of the LpxC band at each time point and normalizing the intensity to a nonspecific band used as a loading control. The level of LpxC at each time point was then presented as a percentage of protein present at time zero for each strain. These data were plotted in GraphPad Prism. The results represent three independent experiments; error bars in the figures show standard deviations (SD).

### Monitoring growth during YejM depletion.

Cultures of TB28/pEMF17 [Δ*lacIZYA*::*frt*/P*_lac_*::*yejM*] and EMF27 [Δ*lacIZYA*::*frt ΔyejM*/P*_lac_*::*yejM*] were grown overnight at 37°C in 4 ml of LB containing Cam plus 50 μM IPTG. Cultures were then diluted to an OD_600_ of 0.025 in 6 ml of LB containing Cam plus 50 μM IPTG and grown at 37°C with shaking to an OD_600_ of 0.3. Then, 3 ml of culture was pelleted for 2 min at 5,000 rpm, washed once in LB containing Cam, and resuspended in 3 ml LB containing Cam. The concentration of the cultures was then normalized, and the samples were diluted at a ratio of 1:50 in 50 LB containing Cam with or without 50 μM IPTG. Cultures were incubated at 37°C with shaking. Samples were taken every 15 to 30 min, as indicated in Fig. 2C. The culture density was measured, and samples of cells were removed for fixation. In order to quantify cell chaining, the number of cellular units per chain (indicated by partial constriction between units) was counted per cell. All data were plotted in GraphPad Prism.

### Effect of CHIR-090 on cell morphology.

MG1655 (WT) cells were grown overnight in LB at 37°C and back diluted to an OD_600_ of 0.05 in 5 ml of LB and grown to an OD_600_ of 0.4. Cells were back diluted again to an OD_600_ of 0.1 in 5 ml LB with dimethyl sulfoxide (DMSO) or 0.25 μg/ml CHIR-090 (Cayman Chemical Company catalog no. 728865-23-4). Cells were grown for 2 h and fixed before being visualized by phase-contrast microscopy. The extent of cell chaining was quantified as described above.

### Phase-contrast microscopy.

For the phase-contrast micrographs in Fig. 1, cells were fixed in 2.6% in formaldehyde and 0.04% glutaraldehyde at room temperature for 1 h and stored at 4°C for up to 3 days. Prior to imaging, samples were immobilized on 2% agarose pads on 1-mm glass slides, and number 1.5 coverslips were used. Samples were imaged on a Nikon TE2000 inverted microscope using Nikon Elements Acquisition software AR 3.2. Cropping was done and additional adjustments were made using Fiji software.

### POLAR analysis.

Cells were prepared for imaging as described previously (33). Briefly, cells from a single colony were grown in LB medium supplemented with Tet and Cam for 2 h at 37˚C. Cells were back diluted in M9 containing 0.2% arabinose and 100 μM IPTG and incubated for 2 h at 37˚C to induce expression of bait and prey protein fusions. Cells were immobilized on agarose pads as described above for imaging. Micrographs were taken on a Nikon Ti inverted microscope with a Plan APO lambda 100×/1.45 oil Ph3 DM lens objective, Lumencore SpectraX LED illumination, Chroma ET filter cubes for GFP (49002) and mCherry (49008), an Andor Zyla 4.2 Plus sCMOS camera, and Nikon Elements 4.30 acquisition software. The microscope slide was kept at 30°C using an environmental control chamber. Demographs showing polar localization were generated using a custom MATLAB code as described previously (33). Cells were aligned by length and oriented so that the pole with greater bait intensity was located on the right. The corresponding demograph of the prey signal was generated using the same orientation.
