# Supplementary figures and images for: An Essential Membrane Protein Modulates the Proteolysis of LpxC to Control Lipopolysaccharide Synthesis in Escherichia coli
Source: mBio. 2020 May 19;11(3):e00939-20. doi: 10.1128/mBio.00939-20 (PMC7240159; doi:10.1128/mBio.00939-20)

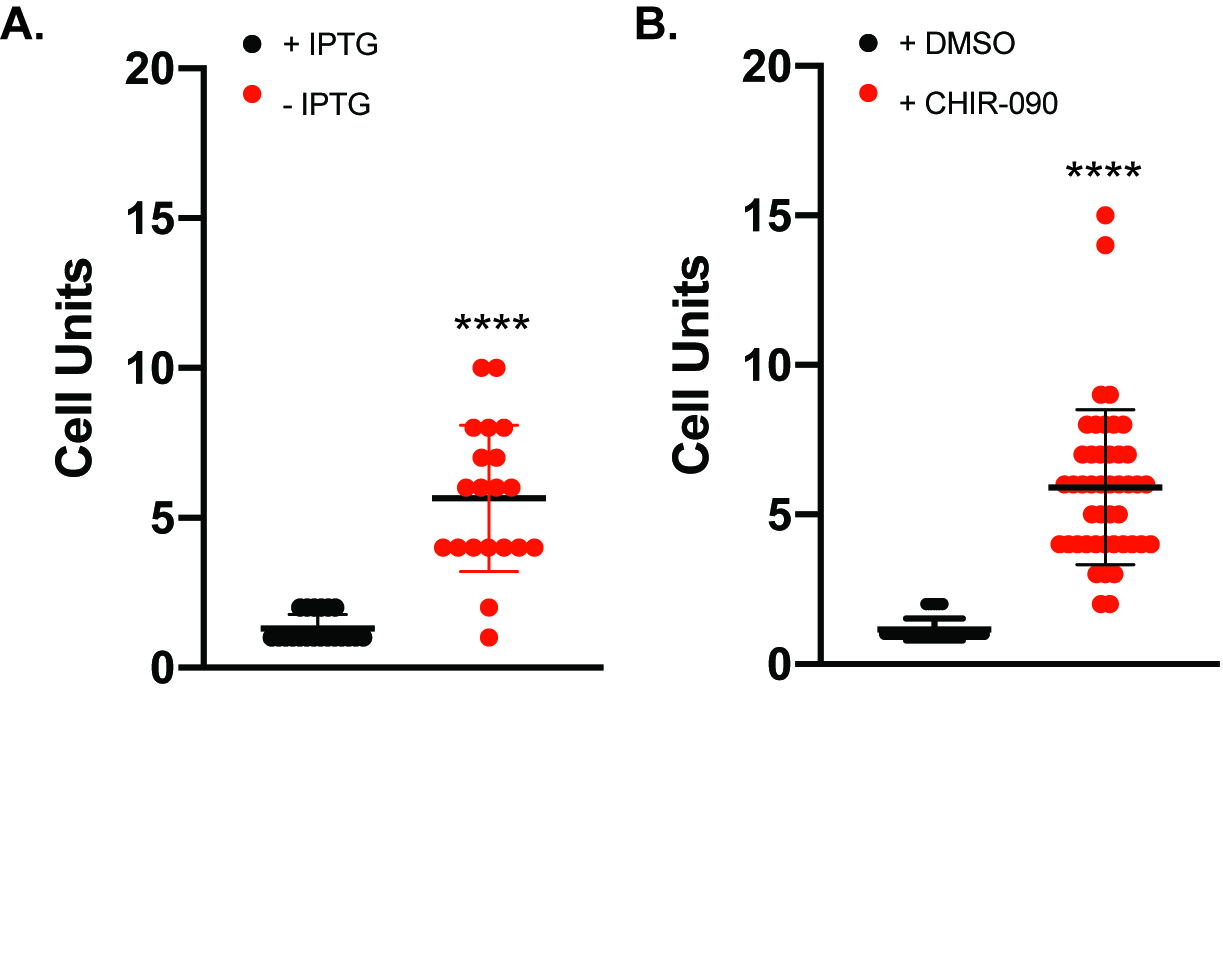

Supplement: FIG S1 [file mBio.00939-20-sf001.tif]

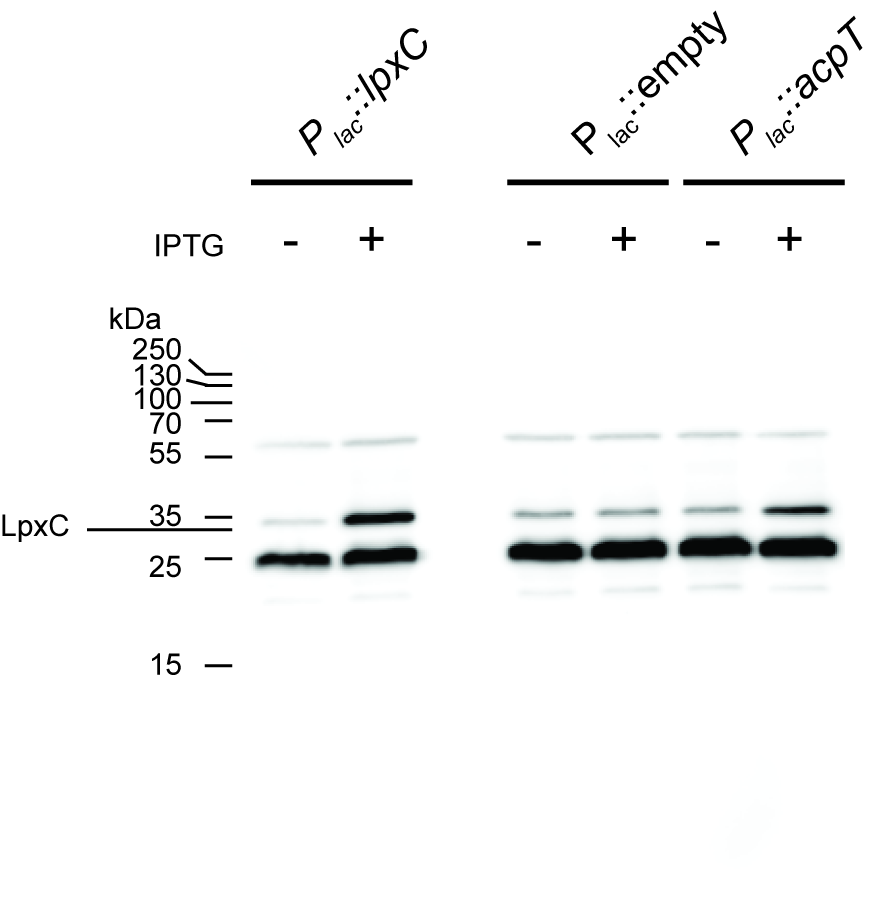

Supplement: FIG S2 [file mBio.00939-20-sf002.tif]

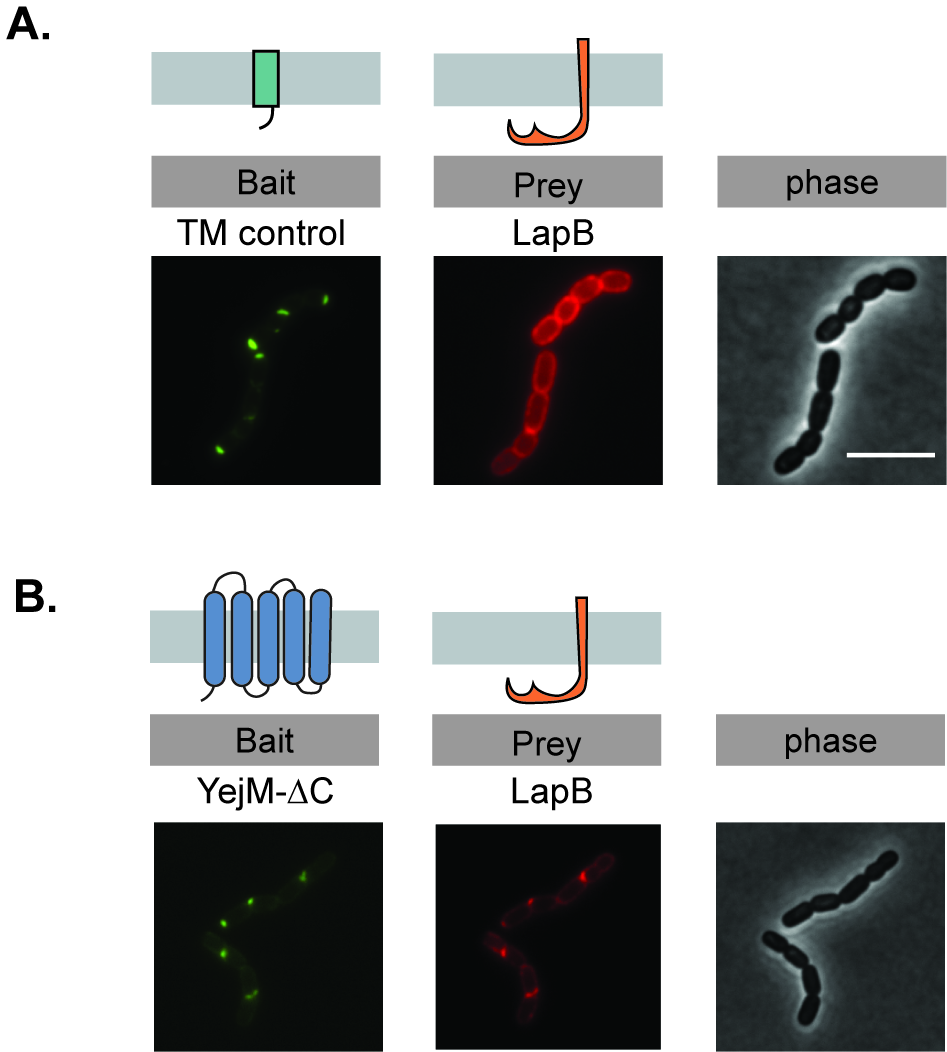

Supplement: FIG S3 [file mBio.00939-20-sf003.tif]
