# Supplementary material for: An Essential Membrane Protein Modulates the Proteolysis of LpxC to Control Lipopolysaccharide Synthesis in Escherichia coli
Source: mBio. 2020 May 19;11(3):e00939-20. doi: 10.1128/mBio.00939-20 (PMC7240159; doi:10.1128/mBio.00939-20)
Supplement: TABLE S1 [file mBio.00939-20-st001.pdf]

Table S1: Δ*yejM* suppressors

Strain	Selection condition	Mutation	Potential causative mutation
EMF40	LB 37˚C	Amplification (*yaiO-yjgX*)	Amplification of *lpxC*
EMF41	LB 37˚C	*aspC(A371A)* (GCT → GCG)<br><br>*lpxC(V37G)* (CAG → CCG)	Point mutant in *lpxC*
EMF42	LB 37˚C	Amplifications (*ykfC-rrlE*) and (*hemG-yhcF*)	Amplification of *lpxC*
EMF43	LB 37˚C	Amplification (*hokE-yjgX*), A → T point mutation upstream of *gspH*	Amplification of *lpxC*
EMF44	LB 37˚C	Amplifications (*hoke-nmpC*) and (*ykfC-mokC*)	Amplification of *lpxC*
EMF45	LB 37˚C 1% SDS	*lapB(H181R)* GTA → GCA	*lapB(H181R)*
EMF46	LB 37˚C 1% SDS	C → A point mutant upstream of *lapAB* operon	C → A point mutant upstream of *lapAB* operon
EMF47	LB 37˚C 1% SDS	*lpxC(L114Q)* *GAC → GTC*	*lpxC(L114Q)*
EMF48	LB 37˚C 1% SDS	*lapA(Q36STOP)* *GTT → ATT*	*lapA(Q36STOP)*
EMF49	LB 37˚C 1% SDS	Amplification (*yejO-yecF*)	unknown
EMF50	LB 37˚C 1% SDS	Amplification (*yejO-yecF*)	unknown

EMF51	LB 37˚C 1% SDS	Amplification (*yaiX*- *yjgX*)	Amplification of *lpxC*
