# Supplementary material for: An Essential Membrane Protein Modulates the Proteolysis of LpxC to Control Lipopolysaccharide Synthesis in Escherichia coli
Source: mBio. 2020 May 19;11(3):e00939-20. doi: 10.1128/mBio.00939-20 (PMC7240159; doi:10.1128/mBio.00939-20)
Supplement: TABLE S2 [file mBio.00939-20-st002.pdf]

Table S2: Strains used in this study

Strain	Genotype[a]	Source/Reference[b]
Dh5a(lpir)	*F- hsdR17 deoR recA1 endA1 phoA supE44 thi-1 gyrA96 relA1 Δ(lacZYA-argF)U169 Ø80dlacZΔM15 λpir*	Laboratory strain
MG1655	rph1 IvG rfb-50	(*1*)
TB28	MG1655 *ΔlacIZYA::frt*	(*2*)
TB10	MG1655 *λΔcro-bio nad::Tn10*	(*3*)
EMF25	TB10 Δ*yejM::kan* + pEMF17	(*λ*Red)/This study
EMF27	TB28 Δ*yejM::kan* + pEMF17	P1(EMF25) X TB28 + pEMF17/This study
EMF30	MG1655 Δ*yejM::kan* +pPR111	P1(EMF25) X MG1655 + pPR111/This study
JLB45	*F- hsdR17 deoR recA1 endA1 phoA supE44 thi-1 gyrA96 relA1 Δ(lacZYA-argF)U169 Ø80dlacZΔM15 λpir ΔlysAR<>tetAR-Psyn135::cI*	Derivative of strain from de Boer lab (*4*)
EMF37	MG1655 Δ*yejM::kan* + pEMF20	P1(EMF25) X MG1655 + pEMF10/This study
EMF40	MG1655 Δ*yejM::kan,* amplification containing *lpxC (yiaO-yjgX)*	This study
EMF41	MG1655 Δ*yejM::kan, lpxC(V37G), aspC(A371A)*	This study
EMF42	MG1655 Δ*yejM::kan* has two amplified regions (*ykfC-rrlE*, *hemG-yhcF*), one of which contains *lpxC*	This study
EMF43	MG1655 Δ*yejM::kan* A -> T mutation upstream of *gspH*, amplification containing *lpxC (hokE-yjgX)*	This study
EMF44	MG1655 Δ*yejM::kan* has two amplified regions, (*hokE-nmpC, ykfC-mokC*) one of which contains *lpxC*	This study
EMF45	MG1655 Δ*yejM::kan, lapB(H181R)*	This study
EMF46	MG1655 Δ*yejM::kan,* C-> A point mutant upstream of *lapAB* operon	This study

EMF47	MG1655 Δ*yejM::kan, lpxC(L114Q)*	This study
EMF48	MG1655 Δ*yejM::ka*n, *lapA(36QSTOP)*	This study
EMF49	MG1655 Δ*yejM::kan,* Amplification (*yejO*-*yecF*)	This study
EMF50	MG1655 Δ*yejM::kan,* Amplification (*yejO*-*yecF*)	This study
EMF51	MG1655 Δ*yejM::kan,* Amplification contains *lpxC*	This study
EMF68	TB10 Δ$yejM_{(1\text{-}191)}$*::kan* + pEMF17	(λRed)/This study
EMF69	TB28 Δ$yejM_{(1\text{-}191)}$*::kan* + pEMF17	P1(EMF68) X TB28 + pEMF17/This study

[a] The kanamycin resistance cassette (*kan*) is flanked by frt sequences for removal by FLP recombinase

[b] Strains generate by P1 transduction are described as follows: P1(donor strain) X recipient strain (See Methods for details)

1. M. Guyer, R. R. Reed, J. Steitz, K. Low, in *Cold Spring Harbor symposia on quantitative biology*. (Cold Spring Harbor Laboratory Press, 1981), vol. 45, pp. 135-140.
2. T. G. Bernhardt, P. A. De Boer, Screening for synthetic lethal mutants in Escherichia coli and identification of EnvC (YibP) as a periplasmic septal ring factor with murein hydrolase activity. *Molecular microbiology* **52**, 1255-1269 (2004).
3. J. E. Johnson, L. L. Lackner, C. A. Hale, P. A. De Boer, ZipA is required for targeting of DMinC/DicB, but not DMinC/MinD, complexes to septal ring assemblies in Escherichia coli. *Journal of bacteriology* **186**, 2418-2429 (2004).
4. B. Liu, L. Persons, L. Lee, P. A. de Boer, Roles for both FtsA and the FtsBLQ subcomplex in FtsN-stimulated cell constriction in E scherichia coli. *Molecular microbiology* **95**, 945-970 (2015).
