# Supplementary material for: An Essential Membrane Protein Modulates the Proteolysis of LpxC to Control Lipopolysaccharide Synthesis in Escherichia coli
Source: mBio. 2020 May 19;11(3):e00939-20. doi: 10.1128/mBio.00939-20 (PMC7240159; doi:10.1128/mBio.00939-20)
Supplement: TABLE S3 [file mBio.00939-20-st003.pdf]

Table S3: Plasmids used in this study

Plasmid	Relevant features[a]	Origin	Reference/source
pKD4	kan^R, Amp^R	R6K	(*1*)
pMT13	CM^R, P_{ara}::*nativeRBS_mreC*	pBR/colE1	
pMT116	Tet^R, attHK, P_{lac}::empty	R6K	(*2*)
pPR66	CM^R, P_{lac}::empty	pBR/colE1	
pPR99	CM^R, P_{ara}::empty	R6K	
pHC514	CM^R, P_{lac}::*slmA*, attL, attI	R6K	
pHC405	CM^R, Para::*artificialRBS_sf-gfp*	pACYC	
pPR111	CM^R, P_{lac}::*nativeRBS_lpxC*	pBR/colE1	This study
pEMF15	CM^R, P_{ara}::*nativeRBS_yejM*	R6K	This study
pEMF17	CM^R, P_{lac}::*native_RBS_ yejM*	pBR/colE1	This study
pEMF33	CM^R, P_{lac}::*artificial_ yejM*	pBR/colE1	This study
pJLB11	*cI857*, *sceI*, P_{lac}::empty	pSC101	Derivative of plasmid from de Boer lab (*3*)
pEMF20	*cI857*, sceI, P_{lac}:: *nativeRBS_ yejM*	pSC101	This study
pHCL147	Tet^R, attHK, P_{lac}:: *ssDsbA-mScarC*	R6K	(*4*)
pHCL149	CM^R , P_{ara}:: *artificialRBS _popZ-H3H4-msfGFP-TM* (N-terminal fusion)	pBR/colE1	(*4*)
pHCL150	CM^R , P_{ara}:: *artificialRBS _popZ-msfGFP-H3H4* (C-terminal fusion)	pBR/colE1	(*4*)
pEMF35	CM^R , P_{ara}:: *artificialRBS _popZ-H3H4-msfGFP-yejM*	pBR/colE1	This study
pEMF36	Tet^R, attHK, P_{lac}:: *artificialRBS_lapB-mScarC*	R6K	This study
pEMF37	Tet^R, attHK, P_{lac}:: *artificialRBS _lapA-mScarC*	R6K	This study

pEMF38	Tet^R, attHK, P_{lac}:: *artificialRBS _ftsH-mScarC*	R6K	This study
pEMF43	CM^R, P_{lac}::*artificialRBS_acpT*	pBR/colE1	This study
pEMF53	Tet^R, attHK, P_{lac}::*artificialRBS_lapB*	R6K	This study
pEMF54	CM^R , P_{ara}::*artificialRBS_ yejM*	pBR/colE1	This study
pEMF55	CM^R , Para::*popZ-FtsH_msfGFP_H3H4*, *artificialRBS_ yejM*	pBR/colE1	This study
pEMF57	CM^R , P_{ara}::*artificialRBS_sf-gfp*	pBR/colE1	This study
pEMF65	CM^R , P_{ara}:: *artificialRBS _popZ-H3H4-msfGFP-yejM$_{(1-191)}$*	pBR/colE1	(*4*)
pEMF68	CM^R , P_{ara}::*artificialRBS_ yejM$_{(1-191)}$*	pBR/colE1	This study

[a] P_{lac} and P_{ara} refer to the lactose and arabinose promoters, respectively. The artificialRBS indicates the RBS of the Φ10 gene from T7 bacteriophage.

1. K. A. Datsenko, B. L. Wanner, One-step inactivation of chromosomal genes in Escherichia coli K-12 using PCR products. *Proceedings of the National Academy of Sciences* **97**, 6640-6645 (2000).
2. N. G. Greene, C. Fumeaux, T. G. Bernhardt, Conserved mechanism of cell-wall synthase regulation revealed by the identification of a new PBP activator in Pseudomonas aeruginosa. *Proceedings of the National Academy of Sciences* **115**, 3150-3155 (2018).
3. B. Liu, L. Persons, L. Lee, P. A. de Boer, Roles for both FtsA and the FtsBLQ subcomplex in FtsN-stimulated cell constriction in E scherichia coli. *Molecular microbiology* **95**, 945-970 (2015).
4. H. C. Lim, T. G. Bernhardt, A PopZ-linked apical recruitment assay for studying protein–protein interactions in the bacterial cell envelope. *Molecular microbiology*, (2019).
