# Supplementary material for: An Essential Membrane Protein Modulates the Proteolysis of LpxC to Control Lipopolysaccharide Synthesis in Escherichia coli
Source: mBio. 2020 May 19;11(3):e00939-20. doi: 10.1128/mBio.00939-20 (PMC7240159; doi:10.1128/mBio.00939-20)
Supplement: TABLE S4 [file mBio.00939-20-st004.pdf]

**Table S4: Primers used in this study**

| Primer name | Sequence | Strain/plasmid |
|---|---|---|
| LpxC_nativeRBS_XbaI5 | CCCCTCTAGATAATTTGGCGAGATAATACGATGATC | pPR111 |
| lpxC_R_HindIII | TGATAAGCTTATTATGCCAGTACAGCTGAAGGCGC | pPR111 |
| YejMKO For | AACGAAGACAAAGCGCACTAAGGGAAACAGATAACAGGTTGTGTAGGCTGGAGCTGCTTC | EMF25, EMF26 |
| YejMKO Rev | GATTGCAAGTAAGATATTTCGCTAACTGATTTATAATTAACATATGAATATCCTCCTTAG | EMF25, EMF26 |
| yejM_kan_seq_F | CCCAACGCCAGGCAATTG | EMF25, EMF26, EMF27, EMF28 |
| yejM _kan_seq_R | TGCGCTACGTGCCGACTC | EMF25, EMF26, EMF27, EMF28 |
| Kan-3’ Out | TTCTATCGCCTTCTTGACGAGTTCTT | EMF25, EMF26, EMF27, EMF28 |
| Kan-5’ Out | GCTTTCTACGTGTTCCGCTTCC | EMF25, EMF26, EMF27, EMF28 |
| LpxC_nativeRBS_XbaI5' | CCCCTCTAGATAATTTGGCGAGATAATACGATGATC | pPR111 |
| lpxC_R_HindIII | TGATAAGCTTATTATGCCAGTACAGCTGAAGGCGC | pPR111 |
| pEMF15_ yejM _xbaI_F | TGGGTCTAGAGGGAAACAGATAACAGGTTATGGTAAC | pEMF15, pEMF17, pEMF18 |
| pEMF15_ yejM _hindIII_R | CGATAAGCTTAAGTAAGATATTTCGCTAACTGATTTATAATTAATC | pEMF15, pEMF17, pEMF18 |
| pEMF16_ yejM _xbaI_F | CAAATCTAGAGGGAAACAGATAACAGGTTATGGTAAC | pEMF16 |
| pEMF16_ yejM _hindIII_R | CGATAAGCTTAAGTAAGATATTTCGCTAACTGATTTATAATTAATC | pEMF16 |
| yejM _F_gibson | GAAACAGCTATGACCATGATTACGAACTCCCGGGGATCTCGATCC | pEMF20 |
| yejM _R_gibson | TGGTACCGTGTCGACTTACTCGAGAAGTAAGATATTTCGCTAACTGATTTATAATTAATC | pEMF20 |

| | | |
|---|---|---|
| xbaI_phi10RBS_ yejM _F | TGGGTCTAGATTTAAGAAGGAGATATACATAT GGTAACTCATCGTCAGCGC | pEMF33, pEMF68 |
| pEMF31_R_hindIII | CGATAAGCTTCTGATTTATAATTAATCAGTTA GCGATAAAACGCTTCTC | pEMF33 |
| pEMF35_F | CGGTGGATCCGTAACTCATCGTCAGCGCTAC | pEMF35, pEMF65 |
| pEMF35_R | TGATAAGCTTATTTATAATTAATCAGTTAGCGA TAAAACGC | pEMF35 |
| pEMF36_F | CCCCTCTAGAAATAATTTTGTTTAACTTTAAGA AGGAGATATACATATGCTGGAGTTGTTGTTTC TG | pEMF36, pEMF53 |
| pEMF36_R | CAGAGGATCCCAGGCCATCAAGACCGCG | pEMF36 |
| pEMF37_F | TATACATATGAAATATTTACTCATTTTCTTACT GGTGTTAG | pEMF37 |
| pEM37_R | CAGAGGATCCTTCCTTCGCCGCTGACGAG | pEMF37 |
| pEMF38_F | CCCCTCTAGAAATAATTTTGTTTAACTTTAAGA AGGAGATATACATATGGCGAAAAACCTAATAC TCTGGC | pEMF38 |
| pEMF38_R | GGACCCTCGAGACCAGAGGATCCCTTGTCGC CTAACTGCTCTG | pEMF38 |
| pEMF40_R | TGATAAGCTTATTACAGGCCATCAAGACCGC | pEMF53 |
| pEMF43_F | CCCCTCTAGATTTAAGAAGGAGATATACATAT GTATCGGATAGTTCTGGGGAAAG | pEMF43 |
| HindIII-acpT-3'R | TACCAAGCTTATCAGTTAACTGAATCGATCCA TTGCAC | pEMF43 |
| pEMF55_F | CGGCCTCGAGTAACTAGCAGGAGATACATAT ATGGTAACTCATCGTCAGCGC | pEMF55 |
| pEMF55_R | TGATAAGCTTAGTCGACTTATCAGTTAGCGAT AAAACGCTTCTC | pEMF55 |
| yejM_truncation_kan _F | ATGTGGTGTATATCTGGGCCTAAGCCAACTT CTATCGCTTGTGTAGGCTGGAGCTGCTTC | EMF68, EMF69 |
| yejM_truncation_kan _R | GATTGCAAGTAAGATATTTCGCTAACTGATTT ATAATTAACATATGAATATCCTCCTTAG | EMF68, EMF69 |
| pEMF65_R | TGATAAGCTTATTTATAATTAATTAGGCCCAG ATATACACCACAT | pEMF65, pEMF68 |
