# Supplementary material for: An Essential Membrane Protein Modulates the Proteolysis of LpxC to Control Lipopolysaccharide Synthesis in Escherichia coli
Source: mBio. 2020 May 19;11(3):e00939-20. doi: 10.1128/mBio.00939-20 (PMC7240159; doi:10.1128/mBio.00939-20)
Supplement: TEXT S1 [file mBio.00939-20-s0001.pdf]

Supplementary Materials for:

Title: An essential membrane protein modulates the proteolysis of LpxC to control lipopolysaccharide synthesis in *Escherichia coli*

Authors: Elayne M. Fivenson[1] and Thomas G. Bernhardt[1,2*]

Affiliations:
[1]Department of Microbiology
Harvard Medical School
Boston, MA 02115

[2]Howard Hughes Medical Institute

*To whom correspondence should be addressed.
Thomas G. Bernhardt, Ph.D.
Harvard Medical School
Department of Microbiology
Boston, Massachusetts 02115
e-mail: thomas_bernhardt@hms.harvard.edu

MATERIALS AND METHODS

Construction of the ΔyejM strain EMF27 [ΔyejM::kan^R/P_{lac}::yejM]

The *kan^R* cassette and flanking FRT sites was amplified from pKD4 using primers with homology domains flanking *yejM* coding sequence (forward primer: AACGAAGACAAAGCGCACTAAGGGAAACAGATAACAGGTTGTGTAGGCTGGAGCTGCTTC, reverse primer: GATTGCAAGTAAGATATTTCGCTAACTGATTTATAATTAACATATGAATATCCTCCTTAG. This PCR product was then transformed into TB10 (*8*) harboring plasmid pEMF17 [P_{lac}::*yejM*]. Transformants were selected on LB + Kan + Cm + IPTG (100 μM). The Δ*yejM::kan^R* allele was confirmed by PCR across the 5' junction using the primers (yejM_kan_seq_F: CCCAACGCCAGGCAATTG, Kan-5' Out: GCTTTCTACGTGTTCCGCTTCC) and 3' junction (yejM_kan_seq_R: TGCGCTACGTGCCGACTC, Kan-3' Out: TTCTATCGCCTTCTTGACGAGTTCTT) and by testing for lack of growth in the absence of inducer. The Δ*yejM::kan^R* allele was then transduced from EMF25 into TB28/pEMF17 to generate strain EMF27. The genotype was confirmed by PCR and lack of growth in the absence of inducer.

Construction of the $yejM_{(1\text{-}191)}$ strain EMF69 [$yejM_{(1\text{-}191)}$::kan^R/P_{lac}::yejM]

The *yejM(1-191)* mutant (referred to as *yejM-ΔC* in main text) was generated by first amplifying the *kan^R* cassette and flanking FRT sites from pKD4 using primers with homology domains designed to introduce a stop codon at residue 192 on the 5' end and flanking the 3' end of the gene (forward primer: ATGTGGTGTATATCTGGGCCTAAGCCAACTTCTATCGCTTGTGTAGGCTGGAGCTG

CTTC, reverse primer:

GATTGCAAGTAAGATATTTCGCTAACTGATTTATAATTAACATATGAATATCCTCCTT AG) This PCR product was then transformed into TB10 (*8*) harboring plasmid pEMF17 [P_{lac}::*yejM*], generating strain EMF68. Transformants were selected on LB + Kan + Cm + IPTG (100 µM). The Δ*yejM::kan*^{R} allele was confirmed by PCR across the 5' junction using the primers (yejM_kan_seq_F: CCCAACGCCAGGCAATTG, Kan-5' Out: GCTTTCTACGTGTTCCGCTTCC) and 3' junction (yejM_kan_seq_R: TGCGCTACGTGCCGACTC, Kan-3' Out: TTCTATCGCCTTCTTGACGAGTTCTT). The Δ*yejM*$_{(1\text{-}191)}$*::kan*^{R} allele was then transduced from EMF68 into TB28/pEMF17 to generate strain EMF69.

Construction of the ΔyejM strain EMF30 [ΔyejM::kan^R/P$_{lac}$::lpxC]

The Δ*yejM::kan*^{R} allele was transduced into MG1655/pPR111 [WT/P_{lac}::*lpxC*]. Transductants were selected on LB + Cam + Kan + 50 µM IPTG. The transduction was confirmed via PCR (see above).

Molecular biology

The polymerase chain reaction (PCR) was conducted using Q5 High fidelity polymerase (New England Biolabs) or GoTaq green master mix (Promega) following manufacturer's protocol. PCR products were purified using the PCR clean up kit from Qiagen or CWBiosciences. Plasmids were isolated using the Miniprep Kit from Qiagen or the plasmid purification kit from CWBiosciences. All plasmids were sequence verified by the DNA Resource Core of Dana-Farber/Harvard Cancer Center.

Plasmid construction

pPR111

LpxC was amplified from gDNA using primers LpxC_nativeRBS_XbaI5' (CCCCTCTAGATAATTTGGCGAGATAATACGATGATC) and lpxC_R_HindIII (TGATAAGCTTATTATGCCAGTACAGCTGAAGGCGC). The resulting insert (xbaI_nativeRBS_lpxC_hindII) was cloned into vector pPR66 using restriction enzymes xbaI and hindIII.

pEMF15

YejM was amplified from gDNA using primers pEMF15_yejM_xbaI_F (TGGGTCTAGAGGGAAACAGATAACAGGTTATGGTAAC) and pEMF15_yejM_hindIII_R (CGATAAGCTTAAGTAAGATATTTCGCTAACTGATTTATAATTAATC). The resulting insert (xbaI_nativeRBS_*yejM*_hindII) was cloned into vector pPR99 using restriction enzymes xbaI and hindIII.

pEMF17

pEMF15 was digested with restriction enzymes xbaI and hindIII-HF and the xbaI_nativeRBS_*yejM*_hindIII insert was gel-extracted (see Materials and Methods) and ligated into digested pPR66.

pEMF43

acpT was amplified from gDNA using primers pEMF43_F (CCCCTCTAGATTTAAGAAGGAGATATACATATGTATCGGATAGTTCTGGGGAAAG) and HindIII-acpT-3'R (TACCAAGCTTATCAGTTAACTGAATCGATCCATTGCAC). The insert (xbaI_artificialRBS_acpT_hindIII) was ligated into pPR66 using using restriction enzymes xbaI and hindIII.

pEMF20

yejM was amplified from pEMF17 using primers yejM_F_gibson (GAAACAGCTATGACCATGATTACGAACTCCCGGGGATCTCGATCC) and yejM_R_gibson (TGGTACCGTGTCGACTTACTCGAGAAGTAAGATATTTCGCTAACTGATTTATAATTAATC) and inserted in pJLB11, which was digested with restriction enzymes smaI and xhoI, via isothermal assembly. This plasmid was constructed in strain JLB45 which expresses the *cI*857, in order to prevent zygotic induction.

pEMF33

yejM was amplified from pEMF17 using primers xbaI_phi10RBS_yejM_F (TGGGTCTAGATTTAAGAAGGAGATATACATATGGTAACTCATCGTCAGCGC) and pEMF31_R_hindIII (CGATAAGCTTCTGATTTATAATTAATCAGTTAGCGATAAAACGCTTCTC). The resulting insert (xbaI_artificialRBS_yejM_hindII) was cloned into vector pPR66 using restriction enzymes xbaI and hindIII.

pEMF35
yejM was amplified from pEMF17 using primers pEMF35_F (CGGTGGATCCGTAACTCATCGTCAGCGCTAC) and pEMF35_R (TGATAAGCTTATTTATAATTAATCAGTTAGCGATAAAACGC). The resulting product (BamHI_*yejM*_hindIII) was cloned into vector pHCL149(*7*) using restriction enzymes BamHI and HindIII-HF.

pEMF36
lapB was amplified from gDNA using primers pEMF36_F (CCCCTCTAGAAATAATTTTGTTTAACTTTAAGAAGGAGATATACATATGCTGGAGTTGTTGTTTCTG) and pEMF36_R(CAGAGGATCCCAGGCCATCAAGACCGCG). The resulting PCR product (xbaI_artificialRBS_lapB_BamHI) was cloned into pHCL147(*7*) using restriction enzymes xbaI and BamHI. The ligation was the transformed into TB28/pTB102 Chung competent cells. The insert was then amplified and sequenced. Correct integration was confirmed via PCR (*9*). The integrated plasmid was then transduced into MG1655. The resulting strain was confirmed via PCR.

pEMF37
lapA was amplified from gDNA using primers pEMF37_F (TATACATATGAAATATTTACTCATTTTCTTACTGGTGTTAG) and pEM37_R (CAGAGGATCCTTCCTTCGCCGCTGACGAG). The resulting product (ndeI_lapA_bamHI) was inserted into pHCL147(*7*) using restriction enzymes ndeI and bamHI. The ligation was the transformed into TB28/pTB102 Chung competent cells. The insert was then amplified and sequenced. Correct integration was confirmed via PCR (*9*). The integrated plasmid was then transduced into MG1655. The resulting strain was confirmed via PCR.

pEMF38
FtsH was amplified from gDNA using primers pEMF38_F (CCCCTCTAGAAATAATTTTGTTTAACTTTAAGAAGGAGATATACATATGGCGAAAAACCTAATACTCTGGC) and pEMF38_R (GGACCCTCGAGACCAGAGGATCCCTTGTCGCCTAACTGCTCTG). The resulting product (xbaI_artificialRBS_ftsH_xhoI) was inserted into pHCL147(*7*) using restriction enzymes xbaI and xhoI. The ligation was the transformed into TB28/pTB102 Chung competent cells. The insert was then amplified and sequenced. Correct integration was confirmed via PCR(*9*). The integrated plasmid was then transduced into MG1655. The resulting strain was confirmed via PCR.

pEMF53
artificalRBS_lapB was amplified from pEMF40 using primers pEMF36_F (CCCCTCTAGAAATAATTTTGTTTAACTTTAAGAAGGAGATATACATATGCTGGAGTTGTTGTTTCTG) and pEMF40_R(TGATAAGCTTATTACAGGCCATCAAGACCGC). The resulting insert (xbaI_artificalRBS_lapB_hindIII) was digested using restriction enzymes xbaI and hindIII-HF and ligated into vector pMT116. The ligation was the transformed into TB28/pTB102 Chung competent cells. The insert was then amplified and

sequenced. Correct integration was confirmed via PCR(*9*). The integrated plasmid was then transduced into MG1655. The resulting strain was confirmed via PCR.

pEMF54
pEMF33 was digested with restriction enzymes xbaI and hindIII-HF. The artificalRBS_yejM insert was ligated into pMT13.

pEMF55
ArtificalRBS_*yejM* sequence was amplified from pEMF35 using primers pEMF55_F(CGGCCTCGAGTAACTAGCAGGAGATACATATATGGTAACTCATCGTCAGCGC) and pEMF55_R(TGATAAGCTTAGTCGACTTATCAGTTAGCGATAAAACGCTTCTC). The resulting insert (xhoI_artificalRBS_*yejM*_hindIII) was digested using restriction enzymes xhoI and hindIII-HF and ligated into vector pHCL149 directly downstream of the Para::popZ-H3H4-msfGFP-TM sequence.

pEMF57
pHC405 was digested with restriction enzymes xbaI and hindIII-HF. The xbaI_sf-gfp_hindIII insert was ligated into pMT13.

pEMF65
yejM was amplified from pEMF17 using primers pEMF35_F (CGGTGGATCCGTAACTCATCGTCAGCGCTAC) and pEMF65_R (TGATAAGCTTATTTATAATTAATTAGGCCCAGATATACACCACAT). The resulting product (BamHI_*yejM(1-191STOP*_hindIII) was cloned into vector pHCL149(*7*) using restriction enzymes BamHI and HindIII-HF.

pEMF68
The *yejM*$_{(1\text{-}191)}$ sequence was amplified from pEMF17 using primers xbaI_phi10RBS_yejM_F (TGGGTCTAGATTTAAGAAGGAGATATACATATGGTAACTCATCGTCAGCGC) and pEMF65_R (TGATAAGCTTATTTATAATTAATTAGGCCCAGATATACACCACAT). The resulting *xbaI_yejM*$_{(1\text{-}191)}$*_hindIII* product was digested using restriction enzymes XbaI and HindIII-HF and ligated into pMT13.

SUPPLEMENTAL REFERENCES

1. M. Guyer, R. R. Reed, J. Steitz, K. Low, in *Cold Spring Harb. Symp. Quant. Biol.* (Cold Spring Harbor Laboratory Press, 1981), vol. 45, pp. 135-140.
2. T. G. Bernhardt, P. A. De Boer, Screening for synthetic lethal mutants in Escherichia coli and identification of EnvC (YibP) as a periplasmic septal ring factor with murein hydrolase activity. *Mol. Microbiol.* **52**, 1255-1269 (2004).
3. J. E. Johnson, L. L. Lackner, C. A. Hale, P. A. De Boer, ZipA is required for targeting of DMinC/DicB, but not DMinC/MinD, complexes to septal ring assemblies in Escherichia coli. *J. Bacteriol.* **186**, 2418-2429 (2004).
4. B. Liu, L. Persons, L. Lee, P. A. de Boer, Roles for both FtsA and the FtsBLQ subcomplex in FtsN-stimulated cell constriction in E scherichia coli. *Mol. Microbiol.* **95**, 945-970 (2015).
5. K. A. Datsenko, B. L. Wanner, One-step inactivation of chromosomal genes in Escherichia coli K-12 using PCR products. *Proceedings of the National Academy of Sciences* **97**, 6640-6645 (2000).
6. N. G. Greene, C. Fumeaux, T. G. Bernhardt, Conserved mechanism of cell-wall synthase regulation revealed by the identification of a new PBP activator in Pseudomonas aeruginosa. *Proceedings of the National Academy of Sciences* **115**, 3150-3155 (2018).
7. H. C. Lim, T. G. Bernhardt, A PopZ-linked apical recruitment assay for studying protein–protein interactions in the bacterial cell envelope. *Mol. Microbiol.*, (2019).
8. D. Yu *et al.*, An efficient recombination system for chromosome engineering in Escherichia coli. *Proceedings of the National Academy of Sciences* **97**, 5978-5983 (2000).
9. A. Haldimann, B. L. Wanner, Conditional-replication, integration, excision, and retrieval plasmid-host systems for gene structure-function studies of bacteria. *J. Bacteriol.* **183**, 6384-6393 (2001).
